# Safety, Tolerability, and Pharmacokinetics of Mirvetuximab Soravtansine in Chinese Patients With Folate Receptor α‐Positive Advanced Ovarian Cancer

**DOI:** 10.1002/cam4.71704

**Published:** 2026-05-21

**Authors:** Yongsheng Li, Li Yuan, Ge Lou, Hongbing Cai, Yuzhi Li, Fenghu Li, Li Wang, Xingtao Long, Yi Gong, Chaonan Zhu, Li Li, June Xu, Qi Zhou

**Affiliations:** ^1^ Chongqing University Cancer Hospital Chongqing China; ^2^ Harbin Medical University Cancer Hospital Harbin China; ^3^ Zhongnan Hospital of Wuhan University Wuhan China; ^4^ The First Affiliated Hospital of Bengbu Medical College Bengbu China; ^5^ Guizhou Provincial Cancer Hospital Guiyang China; ^6^ Henan Cancer Hospital Zhengzhou China; ^7^ Hangzhou Zhongmei Huadong Pharmaceutical Co. Ltd Hangzhou China

**Keywords:** antibody drug conjugate, mirvetuximab soravtansine, refractory ovarian cancer, targeted therapy

## Abstract

**Background:**

Folate receptor α (FRα) is a glycosylphosphatidylinositol‐anchored protein that facilitates folate transport and has emerged as a promising therapeutic target for ovarian cancer, particularly due to its association with tumor progression. This Phase I study investigated the safety, tolerability, pharmacokinetics, and preliminary efficacy of mirvetuximab soravtansine (MIRV), an antibody‐drug conjugate targeting folate receptor α (FRα), in Chinese patients with FRα‐overexpressed platinum‐resistant ovarian cancer.

**Methods:**

This study enrolled 19 Chinese patients with previously treated FRα‐overexpressed ovarian cancer in the dose escalation phase (5 mg/kg, *n* = 4; 6 mg/kg, *n* = 3) and dose expansion phase (*n* = 12). MIRV was administered in escalating doses from 5 to 6 mg/kg (adjusted ideal body weight), following a 3 + 3 dose‐escalation design. The trial is registered with chinadrugtrials.org.cn (CTR20211876).

**Results:**

Median treatment exposure were 9.9 weeks for the 5 mg/kg cohort (*n* = 4) and 13.1 weeks for the 6 mg/kg cohort (*n* = 15). The most common grade ≥ 3 adverse events were decreased platelet count (21.1%), decreased lymphocyte count (15.8%), and anemia (15.8%). The plasma concentration‐time and pharmacokinetic profiles of MIRV and the total antibody were generally comparable after the first and third doses. Pharmacokinetic analysis revealed a median time to maximum concentration (*T*_max_) of 3.33 h, mean terminal half‐life (*T*_1/2_) of 118 h, and geometric mean maximum concentration (*C*_max_) of 137.07 μg/mL. MIRV exposures were comparable to those reported in Caucasian patients. No anti‐drug antibodies were detected. Among 15 efficacy‐evaluable patients with high‐grade serous ovarian cancer, the objective response rate was 26.7%, with partial responses in four patients and stable disease in seven patients.

**Conclusions:**

MIRV showed anticipated pharmacokinetics, safety, tolerability, and efficacy profiles in Chinese patients with FRα‐positive platinum‐resistant ovarian cancers, supporting its potential as a targeted therapeutic agent for this patient population.

**Trial Registration:**

The trial is registered with chinadrugtrials.org.cn (CTR20211876)

## 1 Introduction

Ovarian cancer ranks as the third most common yet the most lethal gynecological malignancy [1]. Annually, both the global incidence and mortality rates of ovarian cancer have been increasing [2], with projections indicating a continuous rise in China from 2019 to 2049 [3]. The lack of specific clinical symptoms and effective biomarkers for screening constitutes a significant barrier to the early detection of ovarian cancer. Consequently, over two‐thirds of diagnosed cases are already at advanced stages or exhibit widespread metastasis, complicating surgical treatment. The survival rate for ovarian cancer is closely linked to the stage of the disease at diagnosis, as well as the tumor histotype and grade [4].

The initial treatment regimen for newly diagnosed advanced ovarian cancer typically includes cytoreductive surgery followed by platinum‐based combination chemotherapy [5]. Although most patients experience clinical relief following this initial treatment, approximately 70% will face recurrence within 2 to 3 years, resulting in a 5‐year survival rate of less than 40% [2, 6]. For managing platinum‐resistant recurrent ovarian cancer (PROC), current therapeutic options primarily involve non‐platinum chemotherapy, administered either as a single agent or in combination with bevacizumab. Most patients with PROC have previously received bevacizumab in earlier treatment lines, leading to the common practice of utilizing sequential single‐agent chemotherapy regimens that include weekly paclitaxel, liposomal doxorubicin, topotecan, etoposide, and gemcitabine [7, 8]. However, the treatment response rates remain dishearteningly low, approaching 10%, with no significant improvement in overall survival rates [7]. In recent years, targeted inhibitors, such as poly (adenosine diphosphate [ADP]‐ribose) polymerase (PARP) inhibitors and epidermal growth factor receptor (EGFR) family‐targeted inhibitors, have demonstrated some benefit in progression‐free survival for specific subpopulations of patients with PROC [9]. Nonetheless, research on most targeted therapies for platinum‐resistant disease has produced unsatisfactory outcomes [10]. Therefore, there is an urgent need to investigate additional treatment options for platinum‐resistant disease to enhance the prognosis for these patients.

Folate receptor α (FRα) plays a crucial role in cell growth and survival, with markedly elevated expression observed in various solid malignant tumors, including non‐small cell lung, ovarian, and endometrial cancers [11]. This overexpression positions FRα as a promising target for therapeutic strategies in these cancer types [11, 12]. Mirvetuximab soravtansine (IMGN853, MIRV), an antibody‐drug conjugate (ADC) developed by ImmunoGen Inc., consists of a humanized anti‐FRα monoclonal antibody linked to the cytotoxic maytansinoid DM4, a potent tubulin disruptor and inhibitor of cell proliferation [13, 14]. Its molecular structure is depicted in Figure S1. Preclinical and clinical studies have demonstrated that MIRV exhibits antitumor activity against FRα‐expressing tumors [13, 15, 16, 17, 18, 19, 20, 21]. Utilizing the regulatory‐approved Ventana FOLR1 (FOLR1‐2.1) companion diagnostic test for patient selection, the SORAYA Phase II single‐arm study and the MIRASOL Phase III randomized controlled trial enrolled patients with platinum‐resistant FRα‐positive advanced ovarian cancer, consistently showed favorable clinical outcomes with MIRV treatment [20, 22]. Based on the positive results of the SORAYA study, MIRV received accelerated approval from the United States Food and Drug Administration (US FDA) in 2022, followed by full approval in 2023 for the treatment of platinum‐resistant FRα‐positive epithelial ovarian, fallopian tube, or peritoneal cancer [11, 23]. The 2024 update of the National Comprehensive Cancer Network (NCCN) guidelines for the management of ovarian, fallopian tube, and peritoneal cancers has since designated single‐agent MIRV as a preferred regimen for this indication [24].

Despite these advances, Asian representation in MIRV clinical trials remains limited, with only 11 Asian patients included among approximately 700 participants enrolled globally, with no ethnicity‐based subgroup analyses reported to date. Given the potential ethnic differences in pharmacokinetics and tolerability, this Phase I was conducted to evaluate the pharmacokinetic properties, safety, and tolerability profiles, along with the preliminary efficacy of MIRV in Chinese patients diagnosed with FRα‐high advanced ovarian and fallopian tube cancers.

## 2 Materials and Methods

This non‐randomized, open‐label Phase I study comprised both dose escalation and dose expansion phases. Conducted across six cancer centers in China, the study adhered to the regulations and guiding principles for ethical medical research established by the China National Medical Products Administration, the International Conference on Harmonization Guidelines for Good Clinical Practice (GCP) E6 (R2), and the World Medical Association Declaration of Helsinki. The protocol and consent forms received approval from the appropriate ethical review boards, and all patients provided written informed consent.

### 2.1 Study Inclusion and Exclusion Criteria

The detailed inclusion and exclusion criteria for the study are outlined in the Supporting Information Methods. In summary, eligible study participants were required to meet the following criteria: (1) Patients aged 18 years or older with FRα‐positive tumors, as confirmed with ≥ 75% of viable tumor cells exhibiting level 2 and/or 3 membrane staining intensity using the Ventana FOLR1 assay (Roche, Basel, Switzerland); (2) those who had previously received at least one line of systemic therapy and lacked other standard treatment options; (3) individuals with at least one measurable lesion, as assessed by the Response Evaluation Criteria in Solid Tumors (RECIST; version 1.1) [3]; (4) patients with an Eastern Cooperative Oncology Group performance status (ECOG PS) of 0 or 1; and (5) individuals demonstrating adequate hematologic, renal, and hepatic function. Key exclusion criteria included peripheral neuropathy of grade 2 or higher, as defined by the National Cancer Institute Common Terminology Criteria for Adverse Events (NCI‐CTCAE; version 5.0); known hypersensitivity to monoclonal antibody or maytansinoid therapy; any active or chronic corneal disorder; a prior clinical diagnosis of treatment‐related pneumonitis; or a history of solid tumor malignancy with a disease‐free interval of less than 3 years, with the exception of adequately treated basal cell or squamous cell skin cancer, in situ cervical cancer, in situ breast cancer, or prostate cancer.

### 2.2 Study Design

This study examined the pharmacokinetic profile of MIRV, administered intravenously as a single agent to eligible Chinese patients with FRα‐positive advanced ovarian tumors. Detailed methodologies for pharmacokinetic analyses are provided in the Supporting Information Methods. Additionally, the secondary objectives of the study include evaluating the safety, tolerability, immunogenicity, and preliminary anti‐tumor efficacy of MIRV.

MIRV was administered via intravenous infusion on Day 1, with treatment cycles repeating every 3 weeks (Q3W). The dosing is determined based on adjusted ideal body weight (AIBW). Phase I studies conducted internationally have established the maximum tolerated dose (MTD) at 6 mg/kg, which is the selected dose for ongoing Phase II and III clinical trials and is also the US FDA‐approved dose for MIRV [13, 15]. In light of the existing safety and efficacy data, a starting dose of 5 mg/kg was chosen for the dose escalation phase of this study, which is lower than the overseas MTD. The dose escalation phase adhered to the standard 3 + 3 escalation design, with one group receiving MIRV at 5 mg/kg (Q3W) and another group receiving 6 mg/kg (Q3W). During cycle 1 (3 weeks), patients in the dose escalation phase were monitored for dose limiting toxicity (DLT). If the two dose groups exhibit comparable safety profiles upon completion of the dose escalation phase. In that case, the higher dose will be selected for the dose expansion phase, which will include the enrollment of an additional 12 patients. The data cut‐off date for this analysis was 22 May 2023.

### 2.3 Pharmacokinetic Assessments

Plasma pharmacokinetics were evaluated during cycle 1 and cycle 3 to estimate several key parameters: maximum concentration (*C*_max_), time to maximum concentration (*T*_max_), minimum concentration (*C*_min_), area under the concentration‐time curve from time 0 to the last measurable concentration (AUC_0‐last_), area under the concentration‐time curve over the dosing interval (AUC_0‐tau_), accumulation ratio based on *C*_max_ (R_ac_*C*max_), and accumulation ratio based on AUC0‐tau (R_ac_AUC0‐tau_). Additionally, when data were available, the area under the concentration‐time curve from time 0 to infinity (AUC_0‐inf_), clearance (CL), half‐life (*t*_1/2_), and volume of distribution (*V*_*d*_) were also calculated.

### 2.4 Immunogenicity Assessments

An immunogenicity confirmatory assay was conducted on samples positive for human anti‐drug antibodies (HADA), and the titers of HADA and neutralizing antibodies (NAb) were subsequently assessed as appropriate.

### 2.5 Clinical Outcome Assessments

Baseline evaluations encompassed a comprehensive medical history and physical examination, ECOG PS blood chemistry and hematology, a serum pregnancy test, an ophthalmologic examination, and electrocardiography. During the screening phase, radiologic imaging was performed using either computed tomography (CT) or magnetic resonance imaging (MRI) of the chest, abdomen, and pelvis. Follow‐up assessments were conducted every 6 weeks (±7 days), employing the same imaging modality utilized at baseline to evaluate overall tumor response according to the RECIST criteria (version 1.1). The objective response rate (ORR) is defined as the proportion of subjects achieving the best response of complete response (CR) or partial response (PR). The disease control rate (DCR) is defined as the proportion of subjects achieving a response (CR or PR) or stable disease (SD) following treatment. The duration of response (DoR) was calculated solely among responders and is defined as the interval from the initial CR or PR until the confirmation of progressive disease (PD). Progression‐free survival (PFS) was calculated as the time from the first dose of MIRV until disease progression or death, whichever occurred first.

### 2.6 Adverse Events (AEs)

AEs are monitored continuously throughout the study, beginning from the administration of the first dose and continuing until 28 days after the termination. AEs are graded according to the NCI‐CTCAE criteria and are recorded using system organ class and preferred terms from the Medical Dictionary for Regulatory Activities (MedDRA).

All patients underwent a comprehensive ophthalmological examination conducted by an ophthalmologist, which included indirect fundoscopy, slit‐lamp examination under pupil dilation, intraocular pressure, and corneal photography. A Schirmer test was performed at screening for all patients, and repeated at the first on‐study ophthalmological examination and as clinically indicated for those with ocular symptoms. Ocular symptoms were assessed prior to each dosing cycle by investigators or other qualified personnel. Patients reporting symptoms were referred for a full ophthalmological evaluation. Those who experience ocular toxicity received complete ophthalmological examinations every other cycle and at the end‐of‐treatment visit, or the 28‐day follow‐up visit after the last dose. Resumption of treatment required resolution of ocular symptoms to Grade ≤ 1 or baseline. If this criterion was not met, dosing was delayed and patients re‐evaluated within 48 to 72 h.

Patients received prophylactic corticosteroid eye drops consisting of 1% prednisolone administered six times daily on Days −1 to 4 and four times daily on Days 5 to 8 of each treatment cycle. For patients intolerant to preservatives, alternative corticosteroids such as difluprednate 0.05% were used per the ophthalmologist's guidance. In addition, preservative‐free artificial tears were used daily, with a minimum 15‐min interval between corticosteroid and lubricating eye drops.

### 2.7 Statistical Analysis

This study did not conduct a formal sample size calculation. Data visualization and analysis were carried out using SAS statistical software (version 9.4), while pharmacokinetic parameters were determined using WinNonlin (version 8.3). Descriptive statistics, including the number of cases, mean with standard deviation, median, minimum, and maximum values, were employed to summarize the data. Pharmacokinetic data were characterized using the geometric mean and coefficient of variation, whereas ordinal data were presented as frequencies and percentages.

## 3 Results

### 3.1 Patient Characteristics

Out of the 88 patients screened for this study, 19 met the inclusion and exclusion criteria and were subsequently enrolled. The dose escalation phase comprised four patients in the 5 mg/kg dose group and three patients in the 6 mg/kg dose group. Following the findings from the dose escalation phase, the 6 mg/kg dose was chosen for the dose expansion phase, which included an additional 12 patients.

This study enrolled 19 female patients of non‐childbearing potential, with a median age of 54 years (range: 45–67 years). The majority of the cohort had an ECOG PS of 1 (17/19, 89.5%), while 2 patients had a PS of 0. All patients, except for one diagnosed with fallopian tube cancer (1/19, 5.3%), had epithelial ovarian cancer (18/19, 94.7%). Each patient exhibited FRα‐positive serous carcinomas, with a predominant occurrence of high‐grade serous carcinoma (16/19, 84.2%). One patient had low‐grade serous carcinoma, and the histological subtype of two patients could not be conclusively identified due to incomplete information. The majority of patients (16/19; 84.2%) had received three or more prior lines of therapy, while the remaining three patients (15.8%) had undergone one or two prior lines of therapy. The median number of prior treatment lines was three (range, 1 to 7). All patients had a history of receiving taxanes (19/19, 100%), with additional treatments including PARP inhibitors (13/19, 68.4%), doxorubicin (12/19, 63.2%), and bevacizumab (11/19, 57.9%). Table 1 provides a summary of the baseline clinical characteristics of the study cohort.

**TABLE 1:** Patient demographics and baseline characteristics.

Characteristics	All (*N* = 19)	MIRV	
		5 mg/kg (*n* = 4)	6 mg/kg (*n* = 15)
Age, year; median (range)	54 (45–67)	48.0 (45–67)	56.0 (48–67)
Sex, *n* (%)			
Female	19 (100.0)	4 (100.0)	15 (100.0)
AIBW; median (range), kg	52.40 (44.6–62.3)	54.40 (51.9–60.3)	50.90 (44.6–62.3)
*Tumor type*			
First diagnosed pathology type, *n* (%)			
Serous carcinoma	19 (100.0)	4 (100.0)	15 (100.0)
Histological grade at initial diagnosis, *n* (%)			
High‐grade	16 (84.2)	3 (75.0)	13 (86.7)
Low‐grade	1 (5.3)	0 (0.0)	1 (6.7)
Undifferentiated	0 (0.0)	0 (0.0)	0 (0.0)
Other	2 (10.5)	1 (25.0)	1 (6.7)
FIGO stage at initial diagnosis, *n* (%)			
I	0 (0.0)	0 (0.0)	0 (0.0)
II	1 (5.3)	0 (0.0)	1 (6.7)
III	8 (42.1)	1 (25.0)	7 (46.7)
IV	3 (15.8)	1 (25.0)	2 (13.3)
Other	7 (36.8)	2 (50.0)	5 (33.3)
ECOG PS score, *n* (%)			
0	2 (10.5)	0 (0.0)	2 (13.3)
1	17 (89.5)	4 (100.0)	13 (86.7)
Number of prior lines systemic treatment, *n* (%)			
1	1 (5.3)	0 (0.0)	1 (6.7)
2	2 (10.5)	1 (25.0)	1 (6.7)
3	7 (36.8)	1 (25.0)	6 (40.0)
4	4 (21.1)	1 (25.0)	3 (20.0)
5	4 (21.1)	1 (25.0)	3 (20.0)
Systemic treatment regimens received^a^, *n* (%)			
Taxanes	19 (100.0)	4 (100.0)	15 (100.0)
PARP inhibitors	13 (68.4)	3 (75.0)	10 (66.7)
Doxorubicin	12 (63.2)	2 (50.0)	10 (66.7)
Bevacizumab	11 (57.9)	1 (25.0)	10 (66.7)
Topotecan	0 (0.0)	0 (0.0)	0 (0.0)

Abbreviations: AIBW, adjusted ideal body weight; ECOG PS, Eastern Cooperative Oncology Group Performance Score; FIGO, The International Federation of Gynecology and Oncology; MIRV, mirvetuximab soravtansine; PARP, poly‐ADP ribose polymerase.

^a^ This lists all treatment regimens received by the patients at different treatment settings and the proportion of patients that received the particular regimen and may not add up to 100%.

MIRV was administered to patients at doses of 5 mg/kg (*n* = 4) and 6 mg/kg (*n* = 15). The dose escalation phase involved seven patients, of whom four received 5 mg/kg and three received 6 mg/kg. The dose expansion phase included 12 patients who were treated with 6 mg/kg.

At the data cut‐off date, all 19 patients had discontinued treatment. Among these, 14 patients (73.7%) discontinued due to disease progression, three patients (15.8%) discontinued due to AEs, and two patients (10.5%) discontinued for other reasons deemed inappropriate for continued treatment by the investigators.

### 3.2 Safety and Tolerability

In the 5 mg/kg dose group, one patient did not complete the DLT observation period; however, all other six patients in the dose escalation cohort completed the DLT observation period without experiencing any DLT events. This outcome suggests that MIRV at doses of 5 mg/kg and 6 mg/kg has an acceptable tolerability profile.

All patients reported at least one treatment‐related adverse event (TRAE), with the most common events (incidence ≥ 30%) being increased aspartate aminotransferase (AST) levels (73.7%), decreased platelet count (68.4%), increased alanine aminotransferase (ALT) levels (63.2%), increased gamma‐glutamyl transferase (47.4%), hypoalbuminemia (42.1%), hypokalemia (31.6%), vomiting (31.6%), and abdominal pain (31.6%) (Table 2). Notably, 11 patients (57.9%) experienced at least one TRAE classified as Grade 3 or higher. The most frequently observed Grade 3 or higher TRAEs (incidence ≥ 10%) included decreased platelet count (21.1%), decreased lymphocyte count (15.8%), anemia (15.8%), keratitis (10.5%), and blurred vision (10.5%). The incidence of other Grade 3 or higher TRAEs was 5.3%, with each of these being single occurrences (Table 2).

**TABLE 2:** Treatment‐related adverse events observed in ≥ 10% of the cohort.

Adverse events	Total (*N* = 19)		5 mg/kg group (*n* = 4)		6 mg/kg group (*n* = 15)	
	All grades	≥ Grade 3	All grades	≥ Grade 3	All grades	≥ Grade 3
At least 1 study drug‐related AE	19 (100.0)	11 (57.9)	4 (100.0)	3 (75.0)	15 (100.0)	8 (53.3)
Investigations	19 (100.0)	8 (42.1)	4 (100.0)	1 (25.0)	15 (100.0)	7 (46.7)
Aspartate aminotransferase increased	14 (73.7)	0	2 (50.0)	0	12 (80.0)	0
Platelet count decreased	13 (68.4)	4 (21.1)	1 (25.0)	0	12 (80.0)	4 (26.7)
Alanine aminotransferase increased	12 (63.2)	0	2 (50.0)	0	10 (66.7)	0
Gamma‐glutamyltransferase increased	9 (47.4)	0	2 (50.0)	0	7 (46.7)	0
Blood alkaline phosphatase increased	5 (26.3)	0	2 (50.0)	0	3 (20.0)	0
Neutrophil count decreased	5 (26.3)	1 (5.3)	1 (25.0)	0	4 (26.7)	1 (6.7)
White blood cell count decreased	5 (26.3)	1 (5.3)	1 (25.0)	0	4 (26.7)	1 (6.7)
Lymphocyte count decreased	5 (26.3)	3 (15.8)	1 (25.0)	1 (25.0)	4 (26.7)	2 (13.3)
Blood urea increased	2 (10.5)	0	0	0	2 (13.3)	0
Weight decreased	2 (10.5)	0	0	0	2 (13.3)	0
Blood glucose increased	2 (10.5)	0	0	0	2 (13.3)	0
Gastrointestinal disorders	13 (68.4)	1 (5.3)	3 (75.0)	1 (25.0)	10 (66.7)	0
Vomiting	6 (31.6)	1 (5.3)	2 (50.0)	1 (25.0)	4 (26.7)	0
Abdominal pain	6 (31.6)	0	1 (25.0)	0	5 (33.3)	0
Nausea	4 (21.1)	0	0	0	4 (26.7)	0
Diarrhea	3 (15.8)	0	1 (25.0)	0	2 (13.3)	0
Abdominal distension	3 (15.8)	0	0	0	3 (20.0)	0
Constipation	2 (10.5)	0	1 (25.0)	0	1 (6.7)	0
Metabolism and nutrition disorders	12 (63.2)	1 (5.3)	2 (50.0)	0	10 (66.7)	1 (6.7)
Hypoalbuminemia	8 (42.1)	0	1 (25.0)	0	7 (46.7)	0
Hypokalemia	6 (31.6)	1 (5.3)	1 (25.0)	0	5 (33.3)	1 (6.7)
Hypomagnesemia	5 (26.3)	0	1 (25.0)	0	4 (26.7)	0
Hypochloremia	4 (21.1)	0	0	0	4 (26.7)	0
Hyperuricemia	3 (15.8)	0	0	0	3 (20.0)	0
Hypertriglyceridemia	3 (15.8)	0	0	0	3 (20.0)	0
Hypoproteinemia	3 (15.8)	0	1 (25.0)	0	2 (13.3)	0
Hypercalcemia	2 (10.5)	0	0	0	2 (13.3)	0
Decreased appetite	2 (10.5)	0	1 (25.0)	0	1 (6.7)	0
Eye disorders	9 (47.4)	3 (15.8)	2 (50.0)	1 (25.0)	7 (46.7)	2 (13.3)
Dry eye	5 (26.3)	0	2 (50.0)	0	3 (20.0)	0
Keratitis	5 (26.3)	2 (10.5)	1 (25.0)	0	4 (26.7)	2 (13.3)
Vision blurred	3 (15.8)	2 (10.5)	0	0	3 (20.0)	2 (13.3)
Nervous system disorders	8 (42.1)	0	0	0	8 (53.3)	0
Hypoesthesia	4 (21.1)	0	0	0	4 (26.7)	0
Peripheral neuropathy	3 (15.8)	0	0	0	3 (20.0)	0
General disorders and administration site conditions	7 (36.8)	1 (5.3)	2 (50.0)	0	5 (33.3)	1 (6.7)
Oedema peripheral	3 (15.8)	0	1 (25.0)	0	2 (13.3)	0
Asthenia	2 (10.5)	0	0	0	2 (13.3)	0
Chest discomfort	2 (10.5)	1 (5.3)	0	0	2 (13.3)	1 (6.7)
Blood and lymphatic system disorders	5 (26.3)	4 (21.1)	2 (50.0)	2 (50.0)	3 (20.0)	2 (13.3)
Anemia	5 (26.3)	3 (15.8)	2 (50.0)	2 (50.0)	3 (20.0)	1 (6.7)
Cardiac disorders	3 (15.8)	0	0	0	3 (20.0)	0
Sinus tachycardia	2 (10.5)	0	0	0	2 (13.3)	0

*Note:* All data are expressed as *n* (%), where n represents the number of subjects. The percentage is calculated by using the number of subjects included in the dataset of each group as the denominator, with the numerator being non‐zero. Each subject is counted only once within the same system organ class (SOC) or preferred term (PT). AEs were coded according to the Medical Dictionary for Regulatory Activities (MedDRA; Chinese version 26.0).

Seven patients (36.8%) experienced at least one serious AE, with three patients (15.8%) reporting serious AEs related to the study drug. Additionally, three patients (15.8%) experienced at least one treatment‐emergent adverse event (TEAE) that led to dose discontinuation due to septic shock, decreased platelet count, and corneal epithelial defect, respectively. Furthermore, two patients (10.5%) reported at least one TEAE that resulted in dose reduction due to decreased neutrophil count, keratitis, and blurred vision, respectively. All of these events were deemed related to the study drug according to the investigator's assessment.

In this cohort, nine patients (47.4%) experienced at least one TEAE of peripheral neuropathy, with eight cases (42.1%) deemed related to the study drug. Notably, pneumonitis was not observed among the participants.

Throughout the study, three patients (15.8%) discontinued treatment due to adverse events, each with distinct clinical profiles and cumulative exposure ranging from two to ten cycles. After receiving six cycles of 6 mg/kg AIBW of MIRV, a patient experienced Grade 4 mixed shock with hemorrhagic components, possibly related to the study drug and septic features attributed to infection. Other contributing factors included prior bevacizumab use and concomitant thrombopoietic agents. A second patient who received ten cycles of 6 mg/kg AIBW of MIRV discontinued treatment following recurrent Grade 3 thrombocytopenia and infusion‐related chest tightness, with underlying coronary artery disease and use of thrombopoietic agents potentially contributing to the toxicity. The third patient received two cycles of 5 mg/kg AIBW of MIRV and developed bilateral Grade 3 corneal epithelial injury despite adherence to prophylactic eye care; this was considered possibly related to treatment. In addition, 11 patients (57.9%) reported either a dose withholding or a delay in dosing. Importantly, no deaths occurred during the study treatment period or within 28 days following the last dose.

### 3.3 Ocular TEAEs

Nine patients (47.4%) experienced at least one ocular TEAE, all of which were associated with the study drug. Eye disorders observed in two patients included dry eye (26.3%), keratitis (26.3%), and blurred vision (15.8%). The remaining ocular TEAEs were isolated occurrences, including cataracts, corneal epithelial defects, glaucoma, retinal drusen, and eye pain. Most patients who experienced ocular AEs either recovered or resolved their symptoms by suspending or delaying their dosing. Among these, three patients (15.8%) experienced at least one ocular TEAE of Grade ≥ 3. One such case is a 67‐year‐old female with recurrent epithelial ovarian cancer who developed Grade 3 bilateral keratitis and blurred vision during cycle 2 of MIRV treatment. Despite strict adherence to prophylactic corticosteroid eye drops, symptoms progressed from Grade 1 to Grade 3 by Day 33, prompting drug interruption. Diagnosis was confirmed via ophthalmological evaluation, with visual acuity declining to 0.12 for the right eye and −0.2 for the left eye. The patient was treated with topical corticosteroids, antibiotics, antivirals, and supportive agents, leading to symptom resolution by Day 66. No further doses were administered due to disease progression. A 45‐year‐old female with recurrent epithelial ovarian cancer who developed bilateral Grade 3 corneal epithelial injury during Cycle 2 of MIRV treatment discontinued dosing, as described above. Despite adherence to prophylactic corticosteroid eye drops, symptoms including photophobia, dacryorrhea, and decreased visual acuity emerged on Day 29, prompting drug discontinuation. Ophthalmological evaluation confirmed injury with visual acuity reduced to 0.2 in the left eye and 0.25 in the right eye. Treatment included topical antibiotics, anti‐inflammatory agents, and lubricants. Symptoms gradually improved over several weeks, with near‐baseline visual acuity restored by Day 72, though full recovery was not confirmed at final assessment. A 52‐ year‐old female with heavily treated epithelial ovarian cancer had a dose reduction from 6 mg/kg to 5 mg/kg AIBW of MIRV after developing Grade 3 blurred vision and keratitis during Cycle 11 of MIRV treatment. Despite consistent use of prophylactic corticosteroid eye drops, keratopathy was confirmed on Day 236 via slit lamp and fluorescein staining. Keratitis was managed with topical recombinant bovine basic fibroblast growth factor gel and deproteinized calf blood extract drops. Vision blurred resolved by Day 266, and keratitis improved to Grade 1 by Day 291, with ongoing ophthalmological monitoring. Treatment was discontinued due to disease progression.

### 3.4 Plasma Pharmacokinetics

**FIGURE 1:**
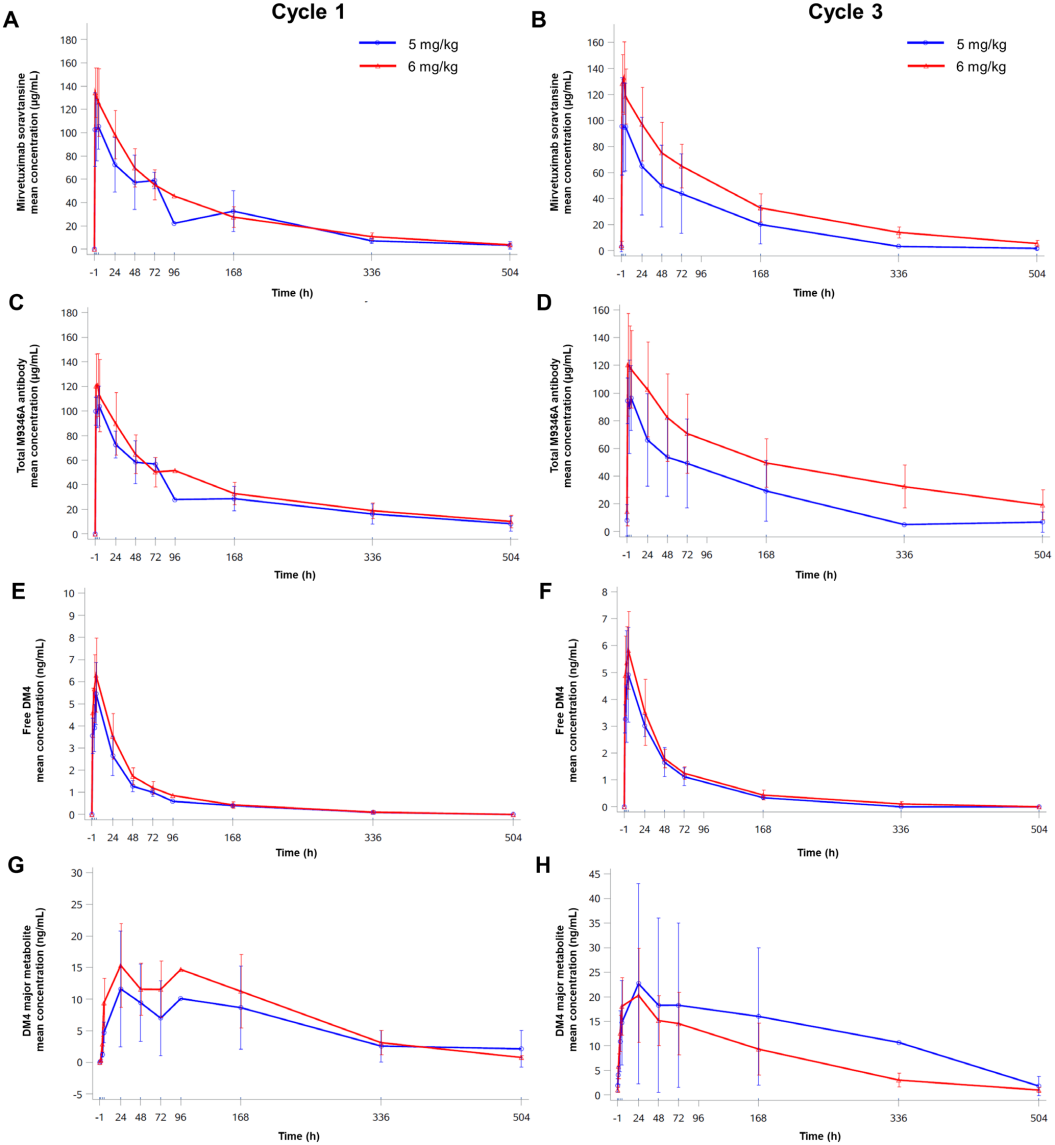
The plasma pharmacokinetic profile of MIRV following both the initial and subsequent doses. The linear plots depict the mean plasma concentration over time during cycles 1 (a, c, e, g) and 3 (b, d, f, h) for MIRV (a, b), the M9346A anti‐FRα antibody (c, d), the free maytansinoid payload (DM4) (e, f), and the major metabolite of DM4 (DM4‐Me) (g, h). The time prior to hour 0 indicates the infusion process, while the time after hour 0 reflects the period following the completion of the infusion.

Pharmacokinetic evaluations were conducted using data from all 19 patients who received MIRV at doses of 5 mg/kg (*n* = 4) and 6 mg/kg (*n* = 15). The plasma concentrations of MIRV, encompassing both conjugated and unconjugated total M9346A antibody, as well as the free payload (DM4) and its primary metabolite (S‐methylated DM4 [DM4‐Me]), were analyzed after the first dose (Cycle 1) and the third dose (Cycle 3). The mean plasma concentrations over time for MIRV, total antibody, free DM4, and DM4‐Me are illustrated in Figure 1, with a summary of the pharmacokinetic parameters presented in Table 3.

**TABLE 3:** Mean plasma pharmacokinetic parameters for MIRV at first dose (Cycle 1) and multiple doses (Cycle 3).

Pharmacokinetic parameters	MIRV				Total M9346A antibody				Free DM4				DM4 major metabolite			
	Cycle 1		Cycle 3		Cycle 1		Cycle 3		Cycle 1		Cycle 3		Cycle 1		Cycle 3	
	5 mg/kg (*n* = 4)	6 mg/kg (*n* = 15)	5 mg/kg (*n* = 2)	6 mg/kg (*n* = 9)	5 mg/kg (*n* = 4)	6 mg/kg (*n* = 15)	5 mg/kg (*n* = 2)	6 mg/kg (*n* = 9)	5 mg/kg (*n* = 4)	6 mg/kg (*n* = 15)	5 mg/kg (*n* = 2)	6 mg/kg (*n* = 9)	5 mg/kg (*n* = 4)	6 mg/kg (*n* = 15)	5 mg/kg (*n* = 2)	6 mg/kg (*n* = 9)
C_max_ (μg/mL; ng/mL†)	109.00 ± 24.50	139.25 ± 25.60	96.65 ± 35.85	137.42 ± 27.06	107.35 ± 10.45	124.97 ± 25.60	98.35 ± 22.13	128.91 ± 29.09	5.46 ± 1.40†	6.35 ± 1.72†	4.92 ± 1.76†	5.87 ± 1.47†	13.89 ± 8.25†	16.81 ± 6.24†	22.87 ± 20.12†	21.64 ± 9.24†
C_last_ (μg/mL; ng/mL†)	12.24 ± 17.84	3.71 ± 1.56	1.70 ± 1.04	5.44 ± 2.48	16.93 ± 17.82	10.20 ± 5.11	6.86 ± 7.41	19.14 ± 10.81	0.51 ± 0.53†	0.18 ± 0.08†	0.34 ± 0.05†	0.19 ± 0.07†	6.06 ± 8.18†	0.78 ± 0.27†	1.83 ± 1.99†	1.01 ± 0.51†
AUC_0‐last_ (h·mg/mL; h·ng/mL†)	10.33 ± 6.57	14.23 ± 3.44	NA	NA	11.93 ± 7.18	16.45 ± 4.36	NA	NA	218.36 ± 25.32†	317.55 ± 79.70†	NA	NA	2042.76 ± 1513.01†	3290.10 ± 1174.99†	NA	NA
AUC_0‐tau_ (h·mg/mL; h·ng/mL†)	NA	NA	10.59 ± 7.11	16.03 ± 4.37	NA	NA	14.42 ± 10.38	23.46 ± 9.07	NA	NA	275.48 ± 67.30†	349.11 ± 68.66†	NA	NA	5450.59 ± 4787.90†	3472.30 ± 1401.43†
AUC_0‐inf_ (h·mg/mL; h·ng/mL†)	13.37 ± 5.76	14.87 ± 3.64	11.20 ± 7.78	17.34 ± 4.85	14.75 ± 8.52	19.95 ± 6.08	17.91 ± 14.95	31.81 ± 14.29	257.34 ± 6.58†	335.95 ± 78.25†	275.91 ± 67.11†	353.24 ± 70.22†	4077.96 ± 4636.61†	3289.66 ± 1284.35†	5823.92 ± 5030.82†	3705.69 ± 1504.45†
*T*_max_ ^a^ (h)	3.96 (1.52–6.15)	3.75 (1.65–6.05)	3.42 (1.15–5.68)	3.33 (1.70–6.88)	5.83 (1.67–6.15)	2.27 (1.72–5.70)	2.49 (1.87–3.12)	2.95 (1.55–5.67)	5.91 (5.52–6.15)	5.70 (3.85–6.17)	5.40 (5.12–5.68)	5.53 (1.55–5.77)	24.51 (6.13–145.52)	26.22 (5.75–336.13)	15.91 (5.12–26.70)	5.77 (5.23–70.40)
*t*½ (h)	103.95 ± 12.34	118.67 ± 11.93	115.16 ± 18.16	140.08 ± 26.71	165.69 ± 99.13	209.07 ± 79.31	174.09 ± 92.74	252.69 ± 78.05	88.39 ± 29.27	74.84 ± 17.77	54.37 ± 6.61	75.32 ± 18.05	299.74 ± 220.21	103.63 ± 14.04	128.44 ± 22.08	123.12 ± 31.94
CL (mL/h)	24.83 ± 13.70	22.64 ± 7.60	36.13 ± 25.64	21.09 ± 5.42	25.16 ± 15.61	17.63 ± 7.44	27.75 ± 20.97	16.02 ± 7.93	NA	NA	NA	NA	NA	NA	NA	NA
*V*_ss_ (L)	3.43 ± 1.44	3.33 ± 0.71	4.89 ± 2.52	3.67 ± 0.63	3.90 ± 0.87	4.46 ± 1.37	5.20 ± 1.18	4.95 ± 1.23	NA	NA	NA	NA	NA	NA	NA	NA
R_ac_AUC0‐tau_	NA	NA	0.97 ± 0.30	1.19 ± 0.25	NA	NA	1.10 ± 0.57	1.44 ± 0.30	NA	NA	1.19 ± 0.22	1.02 ± 0.17	NA	NA	1.54 ± 1.17	0.95 ± 0.20

*Note:* NA denotes parameters were not assessed. For MIRV and Total M9346A antibody, the C_max_ and C_last_ are expressed as μg/mL, and AUC_0‐last_, AUC_0‐tau_, and AUC_0‐inf_ are expressed as h·mg/mL. As denoted by †, C_max_ and C_last_ are expressed as ng/mL and AUC_0‐last_, AUC_0‐tau_, and AUC_0‐inf_ are expressed as h·ng/mL for free DM4 and DM4 major metabolite.

^a^ Data expressed as median (minimum–maximum). All others are presented as mean ± standard deviation.

The plasma concentration‐time and pharmacokinetic profiles of MIRV and the total antibody were generally comparable following both the initial and multiple doses (Figure 1). Systemic exposure, as measured by *C*_max_ and AUC_last_, for both MIRV and the total antibody demonstrated a dose‐proportional increase from 5 to 6 mg/kg. Notably, no significant drug accumulation was observed in Cycle 3 compared to Cycle 1, as indicated by the geometric mean accumulation ratio (R_ac_AUC0‐tau_), which ranged from 0.94 to 1.40. The half‐life in the 6 mg/kg group was approximately 5 days. Additionally, there were no substantial changes in the geometric mean clearance (CL), geometric mean volume of distribution at steady state (Vss), or geometric mean *t*_1/2_ of MIRV between Cycles 1 and 3. Low plasma concentrations of free DM4 and DM4‐Me were detected.

### 3.5 Immunogenicity

Among the 18 patients with at least one post‐baseline HADA result, four were HADA‐positive at baseline and exhibited treatment‐unaffected HADA, characterized by post‐dose titers that were ≤ 4‐fold the baseline titers; three of these patients were transiently positive at baseline. Notably, no patients displayed treatment‐enhanced HADA, defined as a post‐dose titer exceeding 4‐fold the baseline titer. Of the 14 patients who were HADA‐negative at baseline, only one patient showed a single transient HADA‐positive result (titer: 4050) following the administration of the study drug. Across all 19 patients, only one was NAb‐positive (1/19, 5.3%), and this patient was the same individual who exhibited treatment‐emergent HADA positivity. The four patients with treatment‐unaffected HADA were all negative for NAb (Table 4).

**TABLE 4:** Summary analysis of human anti‐drug antibody (HADA).

HADA status	Total (*N* = 19), *n*/*n* (%)	5 mg/kg group (*n* = 4), *n*/*n* (%)	6 mg/kg group (*n* = 15), *n*/*n* (%)
Patients with at least 1 sample tested	19	4	15
Patients with baseline sample tested	19	4	15
Positive	4 (21.05)	1 (25.00)	3 (20.00)
Patients with at least one valid post‐baseline ADA sample tested	18	3	15
Negative → Positive	1 (5.56)	0	1 (6.67)
Positive → Positive (post‐dose titer > 4× baseline titer)	0	0	0
Positive → Positive (post‐dose titer ≤ 4× baseline titer)	4 (22.22)	1 (33.33)	3 (20.00)
Negative → Negative	13 (72.22)	2 (66.67)	11 (73.33)

*Note:* “*n*” refers to the number of subjects within each group of the dataset examined. The notation “*n* (%)” indicates both the actual number of subjects observed and the corresponding percentage. This percentage is calculated by using the number of subjects included in each group's dataset as the denominator, with the numerator being non‐zero. Additionally, the percentage of the population that had at least one valid post‐baseline ADA sample tested is derived from the population that had at least one valid post‐baseline ADA sample as the denominator. In cases where HADA was positive at multiple visits following the first dose, the maximum titer was utilized for the calculations mentioned above. Furthermore, if HADA was positive prior to the first dose but negative thereafter, these results were incorporated into the positive → positive analysis (post‐dose titer ≤ 4 × baseline titer).

### 3.6 Clinical Activity

Following intravenous infusion, the exposure to MIRV increased with escalating doses. In the 5 mg/kg group, the median actual exposure was 9.9 weeks (range: 3.0–37.3 weeks), whereas in the 6 mg/kg group, it was 13.1 weeks (range: 5.9–48.0 weeks). The median number of doses administered was 3.0 (range: 1–11) for the 5 mg/kg group and 4.0 (range: 2–15) for the 6 mg/kg group.

Among the 18 patients evaluable for clinical efficacy, four patients achieved the best objective response of PR, eight patients had SD, and six patients experienced PD, resulting in an ORR of 22.2%. The four responders exhibited a median DoR of 7.3 months (95% CI: 3.8 months, not reached [NR]). Subgroup analysis based on dosage revealed a median DoR of 7.5 months (95% CI: NR, NR) in the 5 mg/kg group and 7.1 months (95% CI: 3.8 months, NR) in the 6 mg/kg group.

A total of 17 patients (89.5%) experienced progression or death following the initiation of the treatment, with a median progression‐free survival (PFS) of 3.9 months (95% confidence interval [CI]: 1.4 to 7.8 months).

Among a cohort of 15 patients with high‐grade serous ovarian cancer who were eligible for efficacy evaluation, the investigators noted PR in four patients and SD in seven patients. The confirmed ORR was calculated to be 26.7% (95% CI: 7.8% to 55.1%), while the DCR was determined to be 73.3% (95% CI: 44.9% to 92.2%). Additionally, the median DoR was reported as 7.3 months (95% CI: 3.8 months to NR), and the median PFS was found to be 4.0 months (95% CI: 1.4 to 7.8 months) as detailed in Table 5.

**TABLE 5:** Objective response of the patients with high‐grade serous ovarian cancer.

Efficacy	Total (*N* = 15), *n* (%)	5 mg/kg group (*n* = 2), *n* (%)	6 mg/kg group (*n* = 13), *n* (%)
Best overall response			
Complete response (CR)	0	0	0
Partial response (PR)	4 (26.7)	1 (50.0)	3 (23.1)
Progressive disease (PD)	4 (26.7)	0	4 (30.8)
Stable disease (SD)	7 (46.7)	1 (50.0)	6 (46.2)
Objective response rate			
Proportion of subjects with objective response	4 (26.7)	1 (50.0)	3 (23.1)
95% Confidence intervals^a^	(7.8, 55.1)	(1.3, 98.7)	(5.0, 53.8)
Disease control rate			
Proportion of subjects with disease control	11 (73.3)	2 (100.0)	9 (69.2)
95% Confidence intervals^a^	(44.9, 92.2)	(15.8, 100.0)	(38.6, 90.9)

^a^ 95% confidence intervals were estimated using the Clopper‐Pearson method.

## 4 Discussion

Our present study assessed the plasma pharmacokinetic profile, safety, tolerability, and preliminary efficacy of MIRV in Chinese female patients with relapsed or refractory FRα‐positive epithelial ovarian cancer and fallopian tube cancer. The majority of patients enrolled in this study had advanced ovarian cancer and experienced disease relapse after multiple lines of therapy.

This study demonstrates that MIRV exhibits good tolerability and a manageable safety profile within the Chinese population. Notably, the drug‐related TRAEs reported in this cohort are consistent with those observed in non‐Chinese patients [13, 15, 16, 17, 18, 20]. The majority of TRAEs were classified as Grade 1 to 2 and were effectively managed using standard care protocols and/or dose adjustments, with only 15.8% of patients discontinuing treatment due to TRAEs. Importantly, increases in ALT (63.2% vs. 18.2%) and AST (73.7% vs. 15.9%) were observed more frequently in the Chinese cohort compared to their non‐Chinese counterparts. The most prevalent Grade ≥ 3 adverse events were myelosuppressive effects, which had not been previously documented. In contrast, low‐grade ocular and gastrointestinal toxicities were the most frequently reported TRAEs in the Caucasian cohort [10, 19].

At a dosage of 6 mg/kg, the *C*_max_ and AUC of MIRV were observed to be 137.07 μg/mL and 13.73 h·mg/mL, respectively, in Chinese patients. In comparison, non‐Chinese patients exhibited Cmax and AUC values of 137.3 μg/mL and 20.65 h·mg/mL, respectively, indicating comparable drug exposure between the two groups [13, 23]. Within our cohort, no DLTs were identified in either the 5 mg/kg or 6 mg/kg groups during the dose escalation phase, leading to the selection of 6 mg/kg for subsequent clinical studies. Treatment with MIRV was associated with a low incidence of HADA and Nab positivity. Notably, no significant increases in antibody titers were observed across all patients following drug administration. Only one patient (1/18, 5.6%) exhibited a single transient instance of HADA positivity after receiving the study drug, while one patient (1/19, 5.3%) developed Nab positivity. The immunogenicity profile of MIRV in the Chinese population was found to be essentially consistent with that observed in the non‐Chinese population [23].

While efficacy was not a primary endpoint of this study and the sample size was limited, treatment with MIRV demonstrated promising clinical outcomes within our cohort. In patients with high‐grade serous ovarian cancer, four individuals achieved a durable PR, resulting in an ORR of 26.7% and a median DoR of 7.3 months. These preliminary results align with findings from the SORAYA study, which reported an ORR of 32.4% and a median DoR of 6.9 months in patients with platinum‐resistant ovarian cancer exhibiting high FRα expression [19]. Similarly, in the Chinese SORAYA bridging study, 35 enrolled patients experienced an ORR of 35.3% and a median DoR of 6.2 months following MIRV treatment [25]. This suggests that the treatment provides comparable clinical benefits and durable responses across different ethnicities. Furthermore, data from the MIRASOL Phase III randomized controlled trial indicated that patients receiving MIRV experienced a 33% reduction in the risk of death compared to those undergoing standard‐of‐care chemotherapy, with median overall survival rates of 16.5 months versus 12.8 months, respectively [20].

In conclusion, the administration of MIRV at doses of up to 6 mg/kg exhibited a pharmacokinetic profile consistent with expectations, alongside acceptable safety and tolerability in Chinese patients diagnosed with platinum‐resistant, FRα‐positive advanced epithelial ovarian cancer and fallopian tube cancer. Additionally, the preliminary clinical efficacy data for MIRV are promising. The consistent results observed in both non‐Chinese and Chinese patient populations regarding the pharmacokinetic, safety, and efficacy profiles of MIRV indicate its potential as a viable treatment option for patients suffering from this specific subset of gynecological malignancies.

## Author Contributions

Qi Zhou, Yongsheng Li, Li Yuan, and June Xu conceived and designed the study. Chaonan Zhu performed the statistical analysis and took responsibility for the integrity of the data and the accuracy of the data analysis. All authors (Yongsheng Li, Li Yuan, Ge Lou, Hongbing Cai, Yuzhi Li, Fenghu Li, Li Wang, Xingtao Long, Yi Gong, Chaonan Zhu, Li Li, June Xu, and Qi Zhou) contributed to the acquisition, analysis, or interpretation of the data. Qi Zhou, Yongsheng Li, Li Yuan, June Xu, and Li Li drafted the manuscript. All authors contributed to the critical revision of the manuscript for important intellectual content, approved the final manuscript, and agreed to be accountable for all aspects of the work.

## Funding

This study is sponsored by Hangzhou Zhongmei Huadong Pharmaceutical Company.

## Ethics Statement

The study was conducted in accordance with the Declaration of Helsinki, with the study protocol approved by the Institutional Ethics Committee of Chongqing University Cancer Hospital (protocol code CZLS2021133‐B and 4 June 2021).

## Consent

Informed consent was obtained from all subjects involved in the study.

## Conflicts of Interest

Chaonan Zhu, Li Li, and June Xu are employees of Hangzhou Zhongmei Huadong Pharmaceutical Company. The other authors declare no known financial interests or personal relationships that could have appeared to influence the work reported in this paper.

## Supporting information


**Figure S1:** Schematic representation of the molecular structure of the antibody‐drug conjugate mirvetuximab soravtansine (MIRV) and its cytotoxic payload. Panel A illustrates the monoclonal antibody component targeting folate receptor alpha (FOLR1) also referred to as M9346A, conjugated to four units of the tubulin inhibitor ravtansine (DM4), a maytansinoid derivative, via lysine residues using the NHS ester group on sulfo‐SPDB. Panel B shows the chemical structure of the DM4 payload and the linker moiety. On average, each M9346A antibody is conjugated to approximately 3.4 DM4 payload molecules.

## Data Availability

Hangzhou Zhongmei Huadong Pharmaceutical Company can provide the data supporting the study findings on request and subject to review.

## References

[1] J. Huang , W. C. Chan , C. H. Ngai , et al., “Worldwide Burden, Risk Factors, and Temporal Trends of Ovarian Cancer: A Global Study,” Cancers 14, no. 9 (2022): 2230, 10.3390/cancers14092230. 35565359 PMC9102475

[2] Institute, NC , Cancer Stat Facts: Ovarian Cancer (National Cancer Institute, 2023).

[3] E. A. Eisenhauer , P. Therasse , J. Bogaerts , et al., “New Response Evaluation Criteria in Solid Tumours: Revised RECIST Guideline (Version 1.1),” European Journal of Cancer 45, no. 2 (2009): 228–247. 19097774 10.1016/j.ejca.2008.10.026

[4] K. Gaitskell , C. Hermon , I. Barnes , et al., “Ovarian Cancer Survival by Stage, Histotype, and Pre‐Diagnostic Lifestyle Factors, in the Prospective UK Million Women Study,” Cancer Epidemiology 76 (2022): 102074. 34942490 10.1016/j.canep.2021.102074 PMC8785125

[5] D. K. Armstrong , R. D. Alvarez , F. J. Backes , et al., “NCCN Guidelines(R) Insights: Ovarian Cancer, Version 3.2022,” Journal of the National Comprehensive Cancer Network 20, no. 9 (2022): 972–980. 36075393 10.6004/jnccn.2022.0047

[6] Clinical Oncology Guidelines Working Committee , Chinese Society of Clinical Oncology (CSCO) Ovarian Cancer Diagnosis and Treatment Guidelines 2020 (Chinese Edition) (2020). People’s Medical Publishing House.

[7] C. Claussen , A. Rody , and L. Hanker , “Treatment of Recurrent Epithelial Ovarian Cancer,” Geburtshilfe und Frauenheilkunde 80, no. 12 (2020): 1195–1204. 33293727 10.1055/a-1128-0280 PMC7714556

[8] J. A. Ledermann , X. Matias‐Guiu , F. Amant , et al., “ESGO‐ESMO‐ESP Consensus Conference Recommendations on Ovarian Cancer: Pathology and Molecular Biology and Early, Advanced and Recurrent Disease,” Annals of Oncology 35, no. 3 (2024): 248–266. 38307807 10.1016/j.annonc.2023.11.015

[9] Q. Guo , Q. Yang , J. Li , G. Liu , I. Nikoulin , and S. Jia , “Clinical Trials of Novel Targeted Therapies in Ovarian Cancer: Moving Beyond Poly ADP Ribose Polymerase (PARP) Inhibitors,” Current Pharmaceutical Biotechnology 19, no. 14 (2018): 1114–1121. 30585545 10.2174/1389201020666181226123054

[10] A. Tattersall , N. Ryan , A. J. Wiggans , E. Rogozinska , and J. Morrison , “Poly(ADP‐Ribose) Polymerase (PARP) Inhibitors for the Treatment of Ovarian Cancer,” Cochrane Database of Systematic Reviews 2, no. 2 (2022): CD007929. 35170751 10.1002/14651858.CD007929.pub4 PMC8848772

[11] T. Gonzalez , M. Muminovic , O. Nano , and M. Vulfovich , “Folate Receptor Alpha—A Novel Approach to Cancer Therapy,” International Journal of Molecular Sciences 25, no. 2 (2024): 1046. 38256120 10.3390/ijms25021046 PMC11154542

[12] I. B. Vergote , C. Marth , and R. L. Coleman , “Role of the Folate Receptor in Ovarian Cancer Treatment: Evidence, Mechanism, and Clinical Implications,” Cancer Metastasis Reviews 34, no. 1 (2015): 41–52. 25564455 10.1007/s10555-014-9539-8

[13] K. N. Moore , H. Borghaei , D. M. O'Malley , et al., “Phase 1 Dose‐Escalation Study of Mirvetuximab Soravtansine (IMGN853), a Folate Receptor Alpha‐Targeting Antibody‐Drug Conjugate, in Patients With Solid Tumors,” Cancer 123, no. 16 (2017): 3080–3087. 28440955 10.1002/cncr.30736 PMC6896318

[14] C. K. Nwabufo , “Mirvetuximab Soravtansine in Ovarian Cancer Therapy: Expert Opinion on Pharmacological Considerations,” Cancer Chemotherapy and Pharmacology 93, no. 2 (2024): 89–105. 37594572 10.1007/s00280-023-04575-y

[15] K. N. Moore , L. P. Martin , D. M. O'Malley , et al., “Safety and Activity of Mirvetuximab Soravtansine (IMGN853), a Folate Receptor Alpha‐Targeting Antibody‐Drug Conjugate, in Platinum‐Resistant Ovarian, Fallopian Tube, or Primary Peritoneal Cancer: A Phase I Expansion Study,” Journal of Clinical Oncology 35, no. 10 (2017): 1112–1118. 28029313 10.1200/JCO.2016.69.9538 PMC5559878

[16] K. N. Moore , I. Vergote , A. Oaknin , et al., “FORWARD I: A Phase III Study of Mirvetuximab Soravtansine Versus Chemotherapy in Platinum‐Resistant Ovarian Cancer,” Future Oncology 14, no. 17 (2018): 1669–1678. 29424243 10.2217/fon-2017-0646

[17] D. M. O'Malley , U. A. Matulonis , M. J. Birrer , et al., “Phase Ib Study of Mirvetuximab Soravtansine, a Folate Receptor Alpha (FRalpha)‐Targeting Antibody‐Drug Conjugate (ADC), in Combination With Bevacizumab in Patients With Platinum‐Resistant Ovarian Cancer,” Gynecologic Oncology 157, no. 2 (2020): 379–385. 32081463 10.1016/j.ygyno.2020.01.037

[18] K. N. Moore , A. M. Oza , N. Colombo , et al., “Phase III, Randomized Trial of Mirvetuximab Soravtansine Versus Chemotherapy in Patients With Platinum‐Resistant Ovarian Cancer: Primary Analysis of FORWARD I,” Annals of Oncology 32, no. 6 (2021): 757–765. 33667670 10.1016/j.annonc.2021.02.017

[19] R. L. Porter and U. A. Matulonis , “Mirvetuximab Soravtansine for Platinum‐Resistant Epithelial Ovarian Cancer,” Expert Review of Anticancer Therapy 23, no. 8 (2023): 783–796. 37458180 10.1080/14737140.2023.2236793

[20] K. N. Moore , A. Angelergues , G. E. Konecny , et al., “Mirvetuximab Soravtansine in FRalpha‐Positive, Platinum‐Resistant Ovarian Cancer,” New England Journal of Medicine 389, no. 23 (2023): 2162–2174. 38055253 10.1056/NEJMoa2309169

[21] O. Ab , K. R. Whiteman , L. M. Bartle , et al., “IMGN853, a Folate Receptor‐Alpha (FRalpha)‐Targeting Antibody‐Drug Conjugate, Exhibits Potent Targeted Antitumor Activity Against FRalpha‐Expressing Tumors,” Molecular Cancer Therapeutics 14, no. 7 (2015): 1605–1613. 25904506 10.1158/1535-7163.MCT-14-1095

[22] R. L. Coleman , D. Lorusso , A. Oaknin , et al., “Mirvetuximab Soravtansine in Folate Receptor Alpha (FRalpha)‐High Platinum‐Resistant Ovarian Cancer: Final Overall Survival and Post Hoc Sequence of Therapy Subgroup Results From the SORAYA Trial,” International Journal of Gynecological Cancer 34, no. 8 (2024): 1119–1125. 38858103 10.1136/ijgc-2024-005401 PMC11347190

[23] Elahere Highlights of Prescribing Information, accessed March, 2023, https://www.accessdata.fda.gov/drugsatfda_docs/label/2022/761310s000lbl.pdf.

[24] Clinical Practice Guidelines in Oncology , “Ovarian Cancer/Fallopian Tube Cancer/Peritoneal Cancer, Version 1.2024,” https://www.nccn.org/guidelines/guidelines‐detail?category=1&id=1453.

[25] L. Li , Y. Wu , Q. Li , et al., “EP323/#463 Efficacy and Safety of Mirvetuximab Soravtansine in Chinese Patients With Platinum‐Resistant Ovarian Cancer With High Folate Receptor Alpha Expression: Results From IMGN853–301 (Soraya China) Study,” International Journal of Gynecological Cancer 33, no. Suppl. 4 (2023): A210.

