# Supplementary material for: Safety, Tolerability, and Pharmacokinetics of Mirvetuximab Soravtansine in Chinese Patients With Folate Receptor α‐Positive Advanced Ovarian Cancer
Source: Cancer Med. 2026 May 21;15(5):e71704. doi: 10.1002/cam4.71704 (PMC13239540; doi:10.1002/cam4.71704)
Supplement: Supplementary file 1 — Figure S1: Schematic representation of the molecular structure of the antibody‐drug conjugate mirvetuximab soravtansine (MIRV) and its cytotoxic payload. Panel A illustrates the monoclonal antibody component targeting folate receptor alpha (FOLR1) also referred to as M9346A, conjugated to four units of the tubulin inhibitor ravtansine (DM4), a maytansinoid derivative, via lysine residues using the NHS ester group on sulfo‐SPDB. Panel B shows the chemical structure of the DM4 payload and the linker moiety. On average, each M9346A antibody is conjugated to approximately 3.4 DM4 payload molecules. [file CAM4-15-e71704-s001.pptx]

## Slide 1
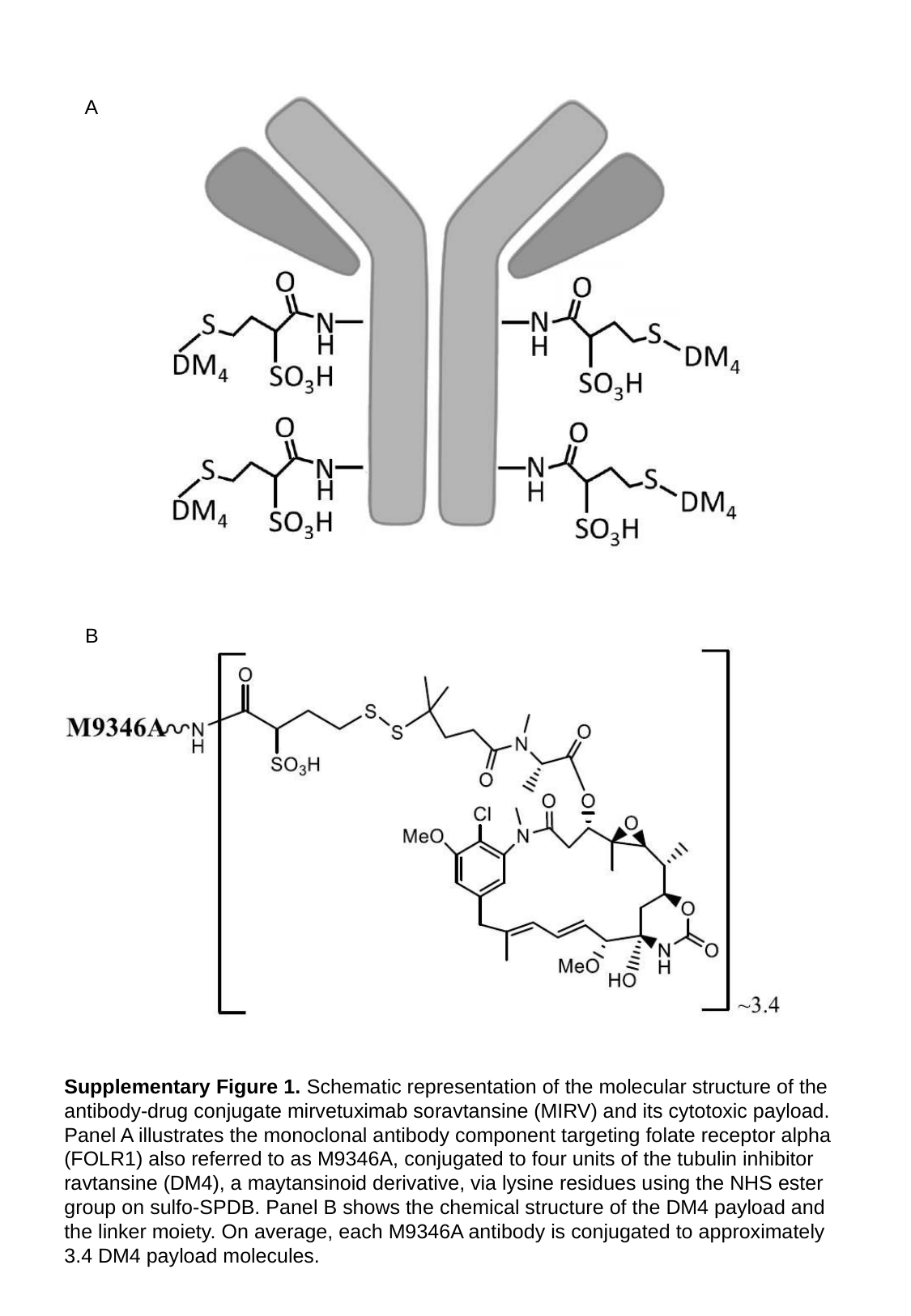

A
B
Supplementary Figure 1. Schematic representation of the molecular structure of the antibody-drug conjugate mirvetuximab soravtansine (MIRV) and its cytotoxic payload. Panel A illustrates the monoclonal antibody component targeting folate receptor alpha (FOLR1) also referred to as M9346A, conjugated to four units of the tubulin inhibitor ravtansine (DM4), a maytansinoid derivative, via lysine residues using the NHS ester group on sulfo-SPDB. Panel B shows the chemical structure of the DM4 payload and the linker moiety. On average, each M9346A antibody is conjugated to approximately 3.4 DM4 payload molecules.
